# Excessive gamma and beta oscillations in manic states across mood and psychotic disorders

**DOI:** 10.1038/s41598-026-40673-6

**Published:** 2026-03-03

**Authors:** Masaya Yanagi, Tsuyoshi Iwasaki, Yoshihiro Iwamura, Osamu Ichikawa, Shizuka Ishida, Osamu Shirakawa, Mamoru Hashimoto, Kazuhito Ikeda.

**Affiliations:** 1 Department of Neuropsychiatry, Faculty of Medicine, Kindai University, 377-2 Ohnohigashi, Osaka-Sayama, Osaka 589-8511 Japan; 2 Research Division, Sumitomo Pharma. Co., Ltd., Suita, Osaka Japan

**Keywords:** Unsupervised learning, Resting-state EEG, ASSR, Bipolar disorder, Major depression, Schizophrenia, Bipolar disorder, Depression, Schizophrenia, Biomarkers, Neurophysiology

## Abstract

The clinical manifestations of mood and psychotic disorders encompass phasic and transdiagnostic features. The complexity of these symptoms may hamper the development of biomarkers for these diagnostic categories. In the present study, machine learning was employed to cluster their transdiagnostic clinical manifestations as a means of developing state-dependent biomarkers with electroencephalography (EEG). This data-driven clustering classified the items of multiple symptom scales into three symptom domains, which subsequently stratified patients with major depressive, bipolar, and schizophrenia spectrum disorders into distinct symptom state groups. The specific brain activity profiles of each stratum were then characterized by resting-state and auditory steady-state EEG paradigms. The resting-state readings of patients in manic states revealed significantly increased gamma and beta oscillations, whereas 40-Hz and 20-Hz auditory steady-state responses (ASSRs) showed no significant differences. These findings suggest that manic states are associated with heightened high-frequency oscillatory activity, represented by gamma and beta oscillations, without a concomitant increase in sensory-evoked information processing precision. Further investigations of excessive gamma and beta oscillations may facilitate the development of state-dependent biomarkers for the assessment, diagnosis, and treatment of manic state symptomatology in clinical psychiatry.

**Supplementary Information:**

The online version contains supplementary material available at 10.1038/s41598-026-40673-6.

## INTRODUCTION

The development of biomarkers has the potential to significantly advance clinical psychiatry. The research domain criteria (RDoC) project has created a framework for biological approaches to psychiatric disorders designed to overcome difficulties in the development of biological therapeutic interventions based on current diagnostic criteria^1^. The identification of biomarkers for mood and psychotic disorders is hindered by the intricate clinical manifestations of these conditions. This includes transdiagnostic clinical symptoms that blur the boundaries between these diagnostic categories^2^. Thus, in bipolar and depressive disorders, psychotic symptoms manifest when affective symptoms become severe^3,4^; while affective symptoms are often apparent in schizophrenia spectrum disorders^5^. Therefore, the symptomatology does not always match the clinical diagnosis precisely. Furthermore, affective and psychotic conditions have significant phase transitions. For example, the transitions between mania and depression in bipolar disorder^3^; and variations in the amount, severity, and persistence of psychotic symptoms in schizophrenia between the prodromal, active, and residual phases^6,7^. These phasic disease courses may hamper the detection of consistent biological findings. In clinical practice, medications such as antipsychotics are prescribed based on more than the diagnostic category alone in recognition of the complexity of psychiatric disease courses^8^. Medication choices are empirically tailored to the transdiagnostic and phasic clinical manifestations of these disorders. However, such flexibility in clinical evaluations and treatments can be subjective, since they often rely on the clinician’s judgment, which is based solely on clinical interviews. To address these issues, there is a need to develop state-dependent biomarkers that can be utilized in the assessment, diagnosis, and treatment of these disorders and their clinical manifestations.

The transdiagnostic symptoms of these psychiatric categories have been biologically substantiated in several large recent population-based and genome-wide studies. This research has shown evidence of correlations between the genetic risks for schizophrenia spectrum, bipolar, and depressive disorders, particularly between bipolar and schizophrenic conditions^9–11^. A recent study has reported the genetic risks of manic and psychotic symptoms to be shared between bipolar disorder and schizophrenia^12^. Furthermore, brain imaging research has found the reduced gray matter volume in the frontotemporal regions^13,14^ and the abnormal connectivity in cerebral white matter^15^ in both schizophrenia and bipolar disorder. Based on the advances in biological research, the Bipolar and Schizophrenia Network for Intermediate Phenotypes Consortium has proposed a biological classification system for psychotic disorders using neuropsychological markers^16,17^. This system is designed to be compatible with psychiatric symptom dimensions rather than the diagnostic categories^2^, allowing for the identification of brain-based biomarkers with the potential to address the transdiagnostic dimensions of clinical symptoms of psychotic disorders.

Electroencephalography (EEG) is a measure of brain function with limited spatial resolution but high temporal resolution. The millisecond (ms) resolution of EEG is able to capture brainwave activity ranging from delta (0.5–4 Hz) to gamma (30–80 Hz) oscillations. A number of resting-state EEG studies of patients with schizophrenia have demonstrated a significantly higher frequency of basal slow wave activity, including delta and theta oscillations, than that found in healthy controls^18^. Similar findings have been reported in studies of patients with bipolar and depressive disorders, although less reportedly than in schizophrenia^18^. Another research of the EEG of patients with schizophrenia has found reduced gamma oscillations in auditory steady-state response (ASSR) readings. These are brainwaves induced by neural entrainment with periodic auditory stimuli, which can probe the ability to generate specific neural oscillations by modulating the frequency of auditory stimuli. In the research showing reduced gamma, the stimuli were delivered at a rate of 40 Hz, which is in the gamma frequency band of oscillations^19^. Similar abnormalities have been reported, to a lesser degree in the 40-Hz ASSR readings of patients with bipolar disorder^20,21^. It may be conjectured that the EEG characteristics shared by these conditions are representative of the transdiagnostic commonalities in symptoms.

**Fig. 1:**
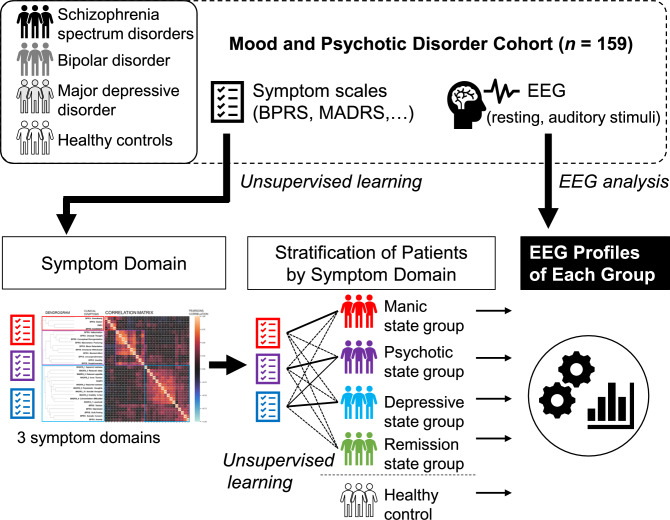
Overview of analysis processes in this study.Multiple symptom scales and EEG data were collected from patients in the mood and psychotic disorder cohort. Symptom domains were defined in a data-driven manner using unsupervised learning from the multiple symptom scales. Based on the three symptom domain scales obtained, patients were stratified into symptom-specific groups through further unsupervised learning. The EEG characteristics of each group were then analyzed to investigate changes in brain activity.

Symptom-based transdiagnostic analyses conducted in a data-driven manner are a promising approach to understanding the phasic and transdiagnostic features of psychiatric disorders, potentially revealing naturally occurring patient subgroups for which novel biomarkers may be identified. In the present study, patients with major depressive, bipolar, and schizophrenia spectrum disorders were stratified by symptom state based on the transdiagnostic symptom domain. The definition of symptom domain and patient stratification were conducted in a data-driven manner using clustering based on unsupervised learning. Then, the resting-state and ASSR EEG readings of these strata were examined to identify their specific brain activity profiles. Figure 1 illustrates the analysis processes in this study. The purpose of this analysis was to characterize the brain activity changes associated with the symptom states of mood and psychotic disorders, facilitating the identification of state-dependent biomarkers by future studies for use in clinical psychiatry.

## Methods

### Participants

A total of 159 Japanese participants were recruited for this study, comprising 41 patients with schizophrenia spectrum disorders (28 with schizophrenia, 10 with schizoaffective disorder, and three with delusional disorders), 49 patients with bipolar disorder, 38 patients with major depressive disorder, and 31 healthy controls. Participants were recruited at Kindai University Hospital, where the majority of patients (95%) were outpatients and the rest were inpatients, and healthy controls were recruited through hospital staff and website. All patients were in a sufficiently stable phase of their disease to be eligible to participate on their own volition. Clinical information on each patient was obtained from the treating clinical psychiatrist and was based on detailed clinical observation over either long-term outpatient treatment or follow-up and/or during hospitalization. Each patient was diagnosed according to the Diagnostic and Statistical Manual (5th edition) (DSM-5) criteria with diagnostic verification by two experienced research psychiatrists. Psychotic symptoms were assessed using the Brief Psychiatric Rating Scale (BPRS)^22^. Manic and depressive symptoms were assessed using the Young Mania Rating Scale (YMRS)^23^ and the Montgomery-Åsberg Depression Rating Scale (MADRS)^24^, respectively. The Snaith-Hamilton Pleasure Scale (SHAPS) was used to evaluate symptoms of anhedonia^25^. Moreover, the Global Assessment of Functioning (GAF) scale was used to assess overall social functioning. The clinical demographic data of participants and mean scores on these clinical scales are shown in Table 1. None of the participants had any history of auditory disorders, neurological disorders, or substance/alcohol abuse. Furthermore, the healthy controls had no history of psychiatric disorders. Each participant was given a complete description of the study before written informed consent was obtained. Anonymization was used to protect all personal information in the data obtained. The study was approved by the Ethics Committee of the Kindai University Faculty of Medicine and the Ethics Committee of Sumitomo Pharma Co., Ltd. It was conducted in accordance with the tenets of the Declaration of Helsinki 1964 and its subsequent amendments.

**Table 1:** Clinical demographics of patients with clinical diagnosis and healthy comparison subjects.

	Diagnostic criteria										
	Schizophrenia spectrum		Bipolar disorder		Major depressive		Healthy control				
	disorders (N = 41 )		(N = 49 )		disorder (N = 38 )		(N = 31 )		Statistics		
	Mean	SD	Mean	SD	Mean	SD	Mean	SD	F	df	p
Age, years	48.0	12.6	51.9	13.6	54.0	14.5	53.0	11.3	1.6	3	0.194
Clinical symptom scores^a^							━	━			
BPRS	14.4	9.3	8.1	6.7	6.5	4.8	━	━	13.6	2	< 0.001
YMRS	1.8	3.2	2.2	4.1	0.7	1.1	━	━	2.5	2	0.085
MADRS	13.5	11.3	11.3	8.9	14.2	9.6	━	━	1.0	2	0.358
SHAPS	1.9	3.0	1.9	2.7	2.0	2.7	━	━	0.0	2	0.997
GAF	50.7	16.3	68.1	18.3	69.8	16.0	━	━	16.0	2	< 0.001
Medications, mg/day											
Antipsychotics^b^	483.5	392.8	152.8	190.2	89.8	218.4	━	━	23.6	2	< 0.001
Lithium	117.1	275.6	549.0	337.3	94.7	257.8	━	━	33.8	2	< 0.001
Antidepressants^c^	22.6	56.4	48.0	90.7	123.1	118.0	━	━	13.0	2	< 0.001
Benzodiazepines^d^	18.5	34.1	8.6	23.4	5.9	9.3	━	━	3.0	2	0.056
	N		N		N		N		χ^2^	df	p
Sex (male/ female)	21 / 20		22 / 27		20 / 18		16 / 15		0.7	3	0.881

^a^BPRS, Brief Psychiatric Rating Scale; YMRS, Young Mania Rating Scale; MADRS, Montgomery-Åsberg Depression Rating Scale; SHAPS, Snaith-Hamilton Pleasure Scale; GAF, Global Assessment of Functioning.

^b^Chlorpromazine equivalent dose.

^c^Imipramine equivalent dose.

^d^Diazepam equivalent dose.

### Symptom domains by data-driven clustering

Using the Python machine learning libraries Scikit-Learn and Scipy^26^, unsupervised machine learning algorithms were applied to identify distinct symptom domains from the items of clinical signs and symptoms. Although the BPRS includes an assessment of psychosis, it is not specifically designed to measure psychotic symptoms alone; therefore, individual subscores were used in this analysis. Given the heterogeneity of depressive symptoms, MADRS subscores were also incorporated. The YMRS is a scale specifically developed to assess manic symptoms. It was included in this study using the total score, as the symptom severity was insufficient to warrant subscore-level analysis. The SHAPS, a unidimensional scale, was also included using its total score. To visualize the relationships and clustering among symptom-scale items, including BPRS, MADRS, SHAPS, and YMRS, Pearson’s correlation coefficients were calculated for each scale item from the 128 patients in our sample with psychiatric diagnoses (i.e., all participants but the healthy controls). Dendrograms were then constructed to visualize the relationships among these symptom-scale items, with more highly correlated items depicted with shorter pairwise branch distances. We used the unweighted average distance method of dendrogram construction with an Euclidean distance metric. Based on these dendrograms, the symptom domains were identified.

### Calculation of the symptom domain scale

A robust scale was defined to capture symptoms across disorders in a data-driven manner by using three distinct symptom domains. The symptom domain scales were calculated for each symptom domain as the means of the z scores for each item on clinical scales.

### Stratification of patients by the symptom domain scales

To stratify patients by the symptom domains, we used the K-means clustering approach based on a distance metric calculated from the symptom domain scales. The hyperparameter, k = 4, which equates to four clusters, was adopted in this study to extract the symptom state groups. Four symptom state groups were named Manic state group, Psychotic state group, Depressive state group, and Remission state group, respectively.

### EEG measurements

EEG measurements of individual participants were performed in a quiet room by a technician on the same day as the clinical assessments. The participant undergoing EEG was instructed to sit and relax on the chair provided, with their eyes closed. To avoid the generation of muscular artifacts, they were also asked to remain motionless. Using a standard EEG device (PolymatePro MP6000, Miyuki Giken, Japan) and compatible active electrodes (AP-C151, Miyuki Giken, Japan), EEG readings were taken from two electrodes placed on points Fp1 and Fp2 of the scalp, according to the international 10–20 system of EEG electrode placement points. These two points correspond to the left (Fp1) and right (Fp2) prefrontal lobes. The reference and ground electrodes were located on bilateral mastoids and another in the middle point between Fz and Fpz (frontal sagittal midline). Prior to electrode placement, the skin surface was wiped with alcohol, and conductive paste (Ten20, Weaver and Company, CO) was applied. When necessary, a skin preparation gel (Nuprep, Weaver and Company, CO) was applied to maintain electrode impedance below 50 kΩ^27,28^. The resting-state EEG of each participant was recorded for 150 s. For the ASSR paradigm, binaural stimuli from an auditory signal processor (RZ6, Tucker-Davis Technologies, Alachua, FL) were presented via an insert phone (TIP-50, Grason-Stadler Inc., Eden Prairie, MN) at an intensity level of 70 dB. The auditory stimuli consisted of 150 blocks of 500 ms click trains alternated with 500 ms inter-train intervals. The click train rates were at 20 or 40 Hz, each presented as a set of 150 blocks. Due to discomfort from the auditory stimuli, two patients (one with bipolar disorder and one with schizophrenia) were unable to complete the 40-Hz auditory stimulation.

### EEG analysis

EEG data were analyzed during resting-state and 40- and 20-Hz ASSRs using previously reported standard methods^29^. EEG readings were filtered using a 0.01–200 Hz band-pass filter and a 60-Hz notch filter. Artifact-free four-second epochs and evoked potential trials were used for power spectrum and ASSR analyses, respectively. Resting-state EEG epochs containing signals exceeding 200 µV were automatically detected and excluded. ASSR recordings were visually inspected for artifact removal by trained technicians. EEG power was calculated with the multitaper method using the MNE-Python package^30^ to analyze the spectral band powers (delta, 0.5–4 Hz; theta, 4–8 Hz; alpha, 8–12 Hz; beta, 12–30 Hz; gamma, 30–80 Hz). ASSR potentials were analyzed for total and evoked powers, and phase locking factor (PLF)^31^. These were calculated using EMSE DataEditor software (Cortech Solutions, Inc., NC) by time-frequency analysis using the Morlet wavelet algorithm at the frequencies 20 Hz (15–25 Hz) and 40 Hz (35–45 Hz) within 500 ms time windows. Induced power was defined as the total power minus the evoked power in the 500-ms periods during auditory stimulation. The value of the evoked power was corrected using the total power at inter-train intervals and used for the subsequent statistical analysis of the evoked power.

### Statistics

All statistical analyses were performed using R 3.6.3 software (https://www.R-project.org/). Analysis of variance and chi-square tests were used, as appropriate, for clinical demographics. Statistical significance was set at *p* = 0.05. For the pairwise group comparisons of EEG profiles, two-sided Tukey’s HSD tests were performed with adjustment for significantly different clinical demographics as covariates. The Bonferroni method, which establishes stringent statistical criteria, was used to search for significant differences in the EEG patterns that characterize each psychiatric state. Because we examined 13 EEG parameters at two brain locations (Fp1 and Fp2), the statistical threshold was set at 0.0019 (0.05/26). The effect size was estimated using Cohen’s *d*.

## RESULTS

### Identification of symptom domains by clustering of transdiagnostic symptoms

**Fig. 2:**
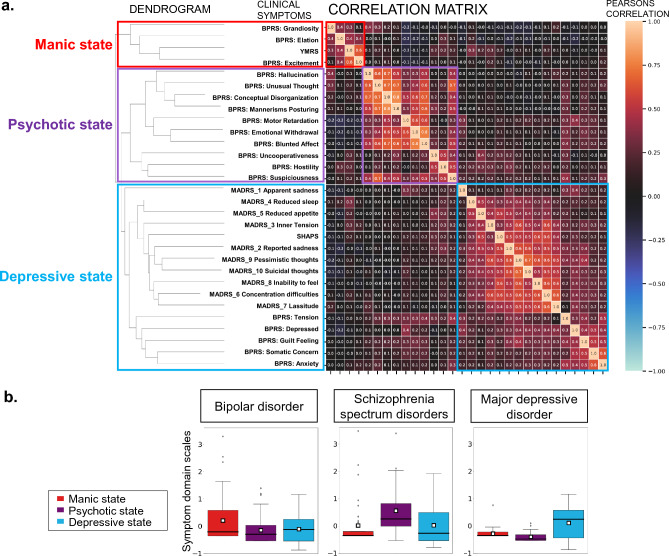
Data-driven clustering of clinical manifestations. Panel (**a**) shows the results of clustering transdiagnostic clinical symptoms into three symptom domains—manic, psychotic, and depressive—using unsupervised machine learning. Note that both positive and negative symptoms were included in the characterization of the psychotic domain. Panel (**b**) shows the distribution of symptom domain scales calculated from multiple clinical scales for the three disorders.

The assessments of patients’ clinical manifestations by multiple clinical scales were clustered using an unsupervised machine learning algorithm. Symptom scores were subjected to hierarchical clustering, and visual inspection of the resulting dendrogram identified a distinct branching point consistent with the classical classification of psychiatric symptom domains. Based on these transdiagnostic dendrograms, items from multiple clinical scales were clustered into three distinct symptom domains: manic, psychotic, and depressive (Fig. 2a). Note that the psychotic domain includes positive and negative symptoms. The symptom domain scales for each diagnostic category are shown in Fig. 2b.

### Stratification of patient psychiatric states

**Fig. 3:**
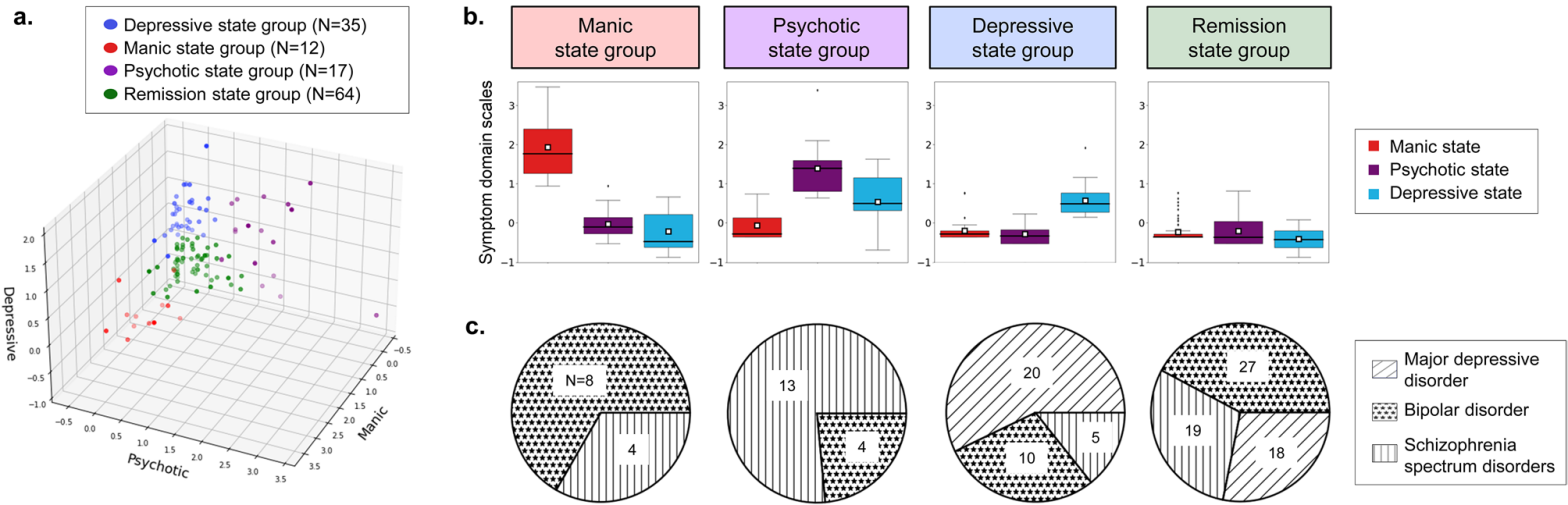
Stratification of patients by the symptom domains. Panel (**a**) plots the data-driven clustering to stratify the patients into four symptom state groups: depressive, manic, psychotic, and remission. Panel (**b**) shows the distribution of symptom domain scales calculated from multiple clinical scales for the four symptom state groups. The manic and depressive state groups were characterized by high scores on the manic and depressive symptom domain scales, respectively. The psychotic state group was characterized by high scores on both the psychotic and depressive symptom domain scales. The remission state group had low scores across all three symptom domain scales. Panel **c** illustrates the distribution of patients’ clinical diagnoses across the four symptom state groups. The patients with bipolar disorder were widely distributed across all four groups, of which the manic state group included primarily patients with bipolar disorder and some patients with schizophrenia spectrum disorders such as schizoaffective disorder.

Subsequently, multiple k values (ranging from 2 to 5) were tested to determine the optimal number of clusters for patient stratification. The resulting cluster structures were evaluated for both clinical relevance and sample size. At k = 2, the data were divided into a manic state group—comprising a limited number of cases—and a broad residual group. Even at k = 3, one cluster encompassed a disproportionately large number of cases, limiting the representation of symptom diversity. Conversely, k = 5 resulted in several clusters with insufficient sample sizes for robust statistical analysis. Based on these evaluations, k = 4 was identified as the most practical and appropriate choice for this study. Accordingly, the patients were stratified into four groups based on the transdiagnostic symptom domains (Fig. 3a). According to their distinct symptom domain profiles, the four symptom state groups were classified as depressive, manic, psychotic, and remission (Fig. 3b). The clinical diagnosis distributions of these four groups are illustrated in Fig. 3c. Details of the clinical variables of the four stratified groups are shown in Table 2.

**Table 2:** Clinical demographics of four groups of psychiatric patients stratified by clinical state.

	Stratification by clinical state										
	Manic state group		Psychotic state group		Depressive state group		Remission state group				
	(N = 12 )		(N = 17 )		(N = 35 )		(N = 64 )		Statistics		
	Mean	SD	Mean	SD	Mean	SD	Mean	SD	F	df	*p*
Age (years)	61.4	10.0	48.3	11.7	46.7	12.6	52.6	14.3	3.5^e^	4^e^	0.009^e^
Clinical symptom scores^a^											
BPRS	10.9	6.1	24.9	6.7	8.8	4.5	5.8	4.2	70.4	3	< 0.001
YMRS	8.0	6.3	1.6	2.3	1.2	1.2	0.7	1.7	30.0	3	< 0.001
MADRS	10.2	9.6	18.4	12.4	22.3	6.4	6.7	4.9	40.6	3	< 0.001
SHAPS	1.3	2.3	3.4	3.9	3.6	3.0	0.8	1.6	12.0	3	< 0.001
GAF	58.2	21.7	42.6	16.3	57.5	15.1	72.4	15.0	18.5	3	< 0.001
Medications, mg/day											
Antipsychotics^b^	295.4	427.3	515.7	444.0	183.6	263.7	187.2	257.8	5.7	3	0.001
Lithium	308.3	334.3	282.4	406.6	237.1	358.2	289.1	369.1	0.2	3	0.904
Antidepressants^c^	9.4	32.5	34.6	72.0	98.9	124.2	59.2	92.5	3.4	3	0.021
Benzodiazepines^d^	21.1	45.6	17.1	21.1	7.7	8.4	9.2	26.6	1.3	3	0.275
	N		N		N		N		χ^2^	df	*p*
Sex (male/ female)	2 / 10		9 / 8		19 / 16		33 / 31		5.7^e^	4^e^	0.220^e^

^a^BPRS, Brief Psychiatric Rating Scale; YMRS, Young Mania Rating Scale; MADRS, Montgomery-Åsberg Depression Rating Scale; SHAPS, Snaith-Hamilton Pleasure Scale; GAF, Global Assessment of Functioning

^b^Chlorpromazine equivalent dose.

^c^Imipramine equivalent dose.

^d^Diazepam equivalent dose.

^e^Comparison of five groups, including four clinical state groups and a healthy control group.

### Resting oscillations and ASSR potentials

Due to insufficient EEG data resulting from incomplete testing or pronounced noise throughout the recordings, four patients (two with schizophrenia, one with bipolar disorder, and one with major depressive disorder) and five patients (two with schizophrenia, two with bipolar disorder, and one with major depressive disorder) were excluded from the EEG analyses during the 40-Hz auditory stimulation and resting-state conditions, respectively. The sample size for each test, reflecting these exclusions, is provided in Supplementary Table 1. The mean epoch rejection rates due to noise in the resting-state EEG were 18.1% ± 20.3% for the healthy control group, 21.0% ± 26.1% for the depressive-state group, 17.5% ± 26.1% for the manic-state group, 19.8% ± 28.9% for the psychotic-state group, and 18.7% ± 25.5% for the remission-state group. No significant differences in the rejection rates were observed among the groups (*F* = 0.24, *p* = 0.92).

**Fig. 4:**
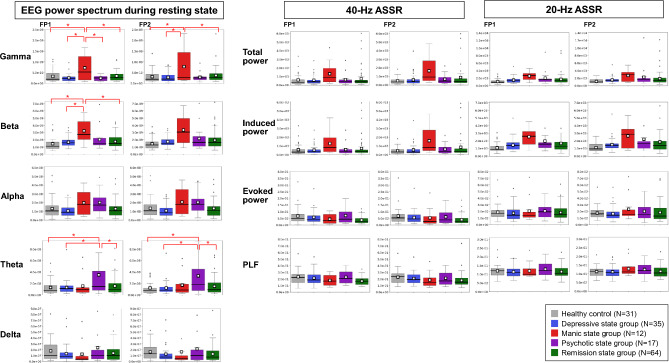
Comparison of the EEG profiles of patients with manic, depressive, or psychotic symptom states, patients in remission, and healthy controls.The left panel shows the EEG power spectrum densities of the different groups during the resting-state recordings. The resting-state paradigm revealed significantly greater gamma oscillations in the manic state group compared to the healthy control, depressive, and remission state groups at both Fp1 and Fp2 sites. The manic state group also exhibited significantly greater gamma oscillations at Fp1 compared to the psychotic state group. Additionally, the manic state group exhibited significantly greater beta oscillations at Fp1 compared to the healthy control, depressive, and remission state groups. For theta oscillations, the psychotic state group exhibited significantly greater activity compared to the healthy control, depressive, and remission state groups at both Fp1 and Fp2 sites. The middle and right panels show the 40-Hz and 20-Hz ASSR potentials across all groups, respectively. No significant differences were detected between groups for either 40-Hz or 20-Hz ASSR at the stringent statistical threshold set in this study. Black horizontal bars indicate median values, and □ indicates mean values. *p < 0.0019.EEG, electroencephalography; ASSR, auditory steady-state response; Fp1, left prefrontal EEG electrode point; Fp2, right prefrontal EEG electrode point; PLF, phase locking factor.

To profile the symptom state-specific brain activities, quantitative resting-state and ASSR EEG data were examined in the stratified symptom states and the healthy control group (Fig. 4). Because significant group differences were found in age, chlorpromazine equivalent doses, and imipramine equivalent doses (Table 2), Tukey tests were conducted that including these confounding factors as covariates. The gamma activity in Fp1 region was significantly higher in the manic state group compared to the healthy control (*p* = 0.0001, *d* = 1.8), remission state (*p* = 0.0002, *d* = 1.5), depressive state (*p* < 0.0001, *d* = 1.8), and psychotic state (*p* = 0.0003, *d* = 1.8) groups. Similarly, the gamma activity in Fp2 region was significantly higher in the manic state group compared to the healthy control (*p* = 0.0004, *d* = 1.4), remission state (*p* = 0.0015, *d* = 1.2), and depressive state (*p* = 0.0003, *d* = 1.5) groups. The beta activity in Fp1 region was significantly higher in the manic state group compared to the healthy control (*p* = 0.0003, *d* = 1.7), remission state (*p* = 0.0005, *d* = 1.4), and depressive state (*p* = 0.0013, *d* = 1.4) groups. The theta activity in Fp1 and Fp2 regions was significantly higher in the psychotic state group compared to the healthy control (*p* = 0.0002 and *p* < 0.0001; *d* = 1.7 and *d* = 1.8, respectively), remission (*p* = 0.0014 and *p* = 0.0007; *d* = 1.2 and *d* = 1.3, respectively), and depressive (*p* = 0.0009 and *p* = 0.0005; *d* = 1.3 and *d* = 1.4, respectively) groups. No significant differences were found in any parameters in either the 40-Hz or 20-Hz ASSR between any of the groups (p > 0.0019). Figure 6a presents the average resting-state EEG power spectra for each group.

### EEG profiles of each diagnostic category

**Fig. 5:**
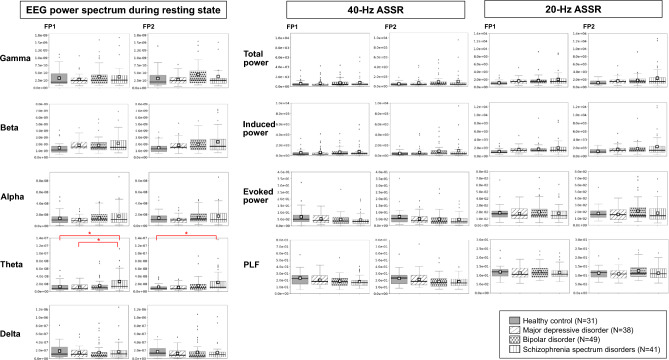
Comparison of the EEG profiles of patients diagnosed with depressive, bipolar, or schizophrenia spectrum disorders and healthy controls.The left panel shows the resting-state power spectrum densities for each group. Patients with schizophrenia spectrum disorder exhibited significantly greater resting-state theta oscillations than healthy controls at both Fp1 and Fp2 sites. Patients with schizophrenia spectrum disorders also had significantly more theta oscillations on Fp1 measurements than those with major depressive disorder. The middle and right panels show the 40-Hz and 20-Hz ASSR EEG measurements, respectively. No significant differences were detected between groups for either 40-Hz or 20-Hz ASSR at the stringent statistical threshold set in this study. The black horizontal bars indicate median values, and □ indicates mean values. *p < 0.0019.

**Fig. 6:**
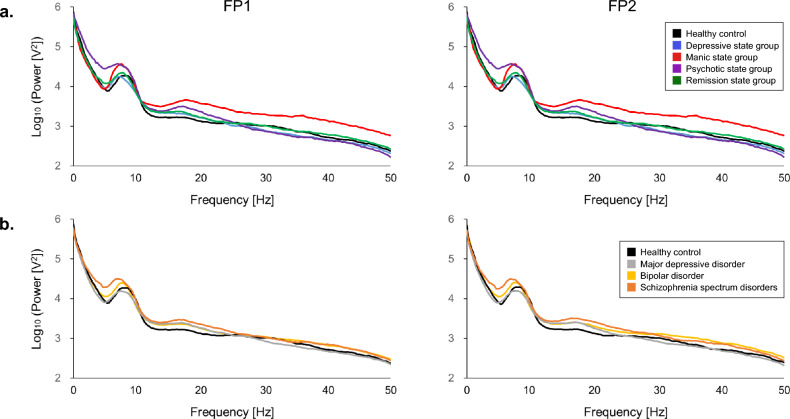
Power spectra plots during resting-state EEG.Panel (**a**) displays the average power spectra across psychiatric state groups stratified by data-driven clustering. Panel (**b**) presents the average power spectra across diagnostic categories.

We found significant differences between the diagnostic category groups in the prescription of chlorpromazine equivalent doses, lithium doses, and imipramine equivalent doses, and a non-significant trend toward differences in diazepam equivalent doses (Table 1). Our Tukey tests, including these confounding factors as covariates, showed that patients with schizophrenia spectrum disorders exhibited a significantly higher resting-state theta activity than healthy controls at Fp1 (*p* = 0.0004, *d* = 1.4) and Fp2 (*p* = 0.0005, *d* = 1.4) regions (Fig. 5). Additionally, the theta activity at Fp1 was significantly higher in patients with schizophrenia spectrum disorders compared to patients with major depressive disorder (*p* = 0.0014, *d* = 1.1). No other significant differences between groups were detected at the stringent statistical threshold set for this study (*p* > 0.0019). Figure 6b presents the average resting-state EEG power spectra across diagnostic categories.

## Discussion

The current study demonstrates the utility of a data-driven approach to the identification of specific brain activity profiles for transdiagnostic symptom states. This study, using a data-driven approach, extracted the three symptoms of mania, depression, and psychosis, which align with the classical diagnostic criteria for mood and psychotic disorders as outlined by Kraepelin (Fig. 2). Our clustering based on the transdiagnostic symptom domains successfully identified patients showing manic symptoms among a group diagnosed with depressive, bipolar, and schizophrenia spectrum disorders (Fig. 3). The EEG profiling based on this clustering identified an excess of gamma and beta activity in the manic state group (Fig. 4), whereas that based on the diagnostic category confirmed the previously established finding of increased theta activity in patients with schizophrenia^18^ (Fig. 5).

The gamma and beta frequency bands are thought to be involved in the cortical processing of functional sensorimotor information^32^. Therefore, the significantly elevated activity in these bands observed in the manic state group suggests that patients in manic states have enhanced information processing during resting state. In contrast, there was no significant increase in either the evoked power or PLF of the manic state group beyond that seen in other groups in either the 40-Hz or 20-Hz ASSR EEG. The 40-Hz and 20-Hz ASSRs assess neural responses in the gamma and beta ranges, respectively. The evoked power and PLF index represent the phase-locked response to the periodic auditory stimuli in ASSR, in terms of magnitude and phase consistency, respectively^33^. The lack of significant increases in evoked power and PLF suggest that the precise neural response to the auditory stimuli was not enhanced in the manic state group. We can infer that cortical information processing, manifesting as high-frequency oscillations, is enhanced in manic states but with no corresponding increase in sensory-evoked information processing precision. This heightened but imprecise information processing might reflect a biological basis for manic symptoms, such as pressured speech, hyperactivity, and flight of ideas.

In contrast to the more complex structure of the psychotic state group, the manic state group was distinguishable, as evidenced by its separation at the k = 2 level in the data-driven clustering. The psychotic state encompassed both positive and negative symptoms, which may explain why the increase in theta oscillations observed in schizophrenia spectrum disorders was also evident in the psychotic state group. These results indicate the need for larger sample sizes and more detailed clinical assessments to better differentiate the complex clinical manifestations of these symptom states. Nonetheless, increased gamma activity was observed in the manic state group, despite the limited sample size. Rodent studies have shown that ketamine, an NMDA receptor antagonist, induces hyperlocomotive activity and significantly increased gamma oscillations^34,35^. Similarly, the human brain is known to respond to ketamine with increased gamma oscillations^36–38^. Recent clinical research has demonstrated antidepressant effects of ketamine in patients with depressive disorders^39^. The antidepressant effects of ketamine could be attributed to the increase in gamma activity, particularly in patients with depressive disorders who exhibited low baseline gamma activity before ketamine administration^38,40^. Thus, the excessive gamma activity observed in the manic group in this study contrasts with the gamma reduction reported in depressive disorders, where ketamine exerted its antidepressant effects, suggesting that gamma activity may vary bidirectionally across the affective spectrum. The role of high frequency oscillations, gamma, in particular, warrants further investigation to determine their relationship to affective alterations in human and animal disease models.

This study also demonstrated that resting beta activity was higher in the manic state group compared to the depressive, remission, and control groups; however, further validation in a larger sample is warranted. Previous studies have reported conflicting regarding the resting-state beta oscillations in patients with bipolar disorder. Two studies have reported significantly higher activity of resting-state beta in patients with bipolar disorder; one in comparison to healthy controls^41^ and one in comparison to patients with schizophrenia^42^. However, two other studies found no significant difference in the beta activity in patients with bipolar disorder compared to healthy controls^43,44^. This discrepancy could be related to variations in the symptom states of the patients in these studies, with bipolar patients, having the potential to be in opposite arousal states depending on the phase of their illness (mania or depression). Our preliminary findings suggest increased beta oscillations in the manic state, implying that the detectability of higher beta activity in the bipolar cohort may partly depend on the proportion of patients in the manic state.

The psychiatric state of each patient who participated in this study was sufficiently stable to allow them to sit still during the EEG measurements. This will have created a degree of selection bias, in that the manic states captured would have been mild. This was confirmed by the manic symptom scale (YMRS) scores of the manic state group, which were not very high (mean scores of 8.0) (see Table 2). Detecting hypomania is important as early intervention can prevent its progression to full-blown mania^45^. However, detection can be difficult through clinical interviews alone, as patients often fail to recognize hypomanic episodes, making input from close relatives essential. Therefore, identifying objective EEG patterns associated with hypomania could provide substantial benefits for both clinicians and patients by aiding in the recognition of hypomanic states and thereby potentially improving early intervention rates for mania.

This study had some limitations. First, we recorded EEG only from two frontal lobe locations (Fp1 and Fp2 in the international 10–20 system). Increases in theta and delta activity across broad cortical regions, including the frontal Fp areas, have been reported in patients with schizophrenia in resting-state EEG recordings^41,46–48^. In contrast, alterations in ASSR are more commonly observed at central sites, such as Fz and Cz^20,21,31,49^. Accordingly, the potential influence of electrode placement on the study’s results cannot be entirely ruled out. Additionally, the influence of minor muscle activity and microsaccades on these two electrodes cannot be fully dismissed. Second, the sample size in this study was limited, with the manic state group comprising particularly few participants. A conservative Bonferroni correction was applied to minimize the risk of Type I errors. However, given the likely correlation between Fp1 and Fp2, this correction may have increased the risk of Type II errors, potentially obscuring other significant findings. Third, as this was a cross-sectional study, we could not examine the recoverability of excessive gamma and beta oscillations. Longitudinal studies are required in the future to verify whether excessive gamma and beta oscillations are improved through the amelioration of manic states.

In this study, the transdiagnostic dissection of patients with depressive, bipolar, and schizophrenia spectrum disorders revealed excessive gamma and beta oscillations in the EEG of patients in manic states. It is hoped that this finding may contribute to the identification of state-dependent biomarkers for the assessment, diagnosis, and treatment of these patients. In addition to EEG, the use of multiple modalities, such as structural brain imaging, may provide a comprehensive picture of state- and trait-dependent effects. Further research with these objectives would address the phasic and transdiagnostic clinical manifestations of mood and psychotic disorders in biologically reconstructing them.

## Supplementary Information

Supplementary Information.

## Data Availability

The data supporting the findings of this study are available from the corresponding author upon reasonable request.
